# Supplementary material for: Mac-2 binding protein glycosylation isomer, the FIB-4 index, and a combination of the two as predictors of non-alcoholic steatohepatitis
Source: PLoS One. 2022 Nov 10;17(11):e0277380. doi: 10.1371/journal.pone.0277380 (PMC9648718; doi:10.1371/journal.pone.0277380)
Supplement: Attachment — Submitted filename: Comments for Manuscript Number PONE-D-22-23754.docx [file pone.0277380.s001.docx]

In this study, Ito et al. evaluates Mac-2 binding protein glycosylation isomer and the FIB-4 index as biomarkers for NASH diagnosis. They find that a combination of these two factors can accurately predict NASH. Although a very interesting study, there are some concerns:

- **Abstract:** There are some grammatical issues.
  - The word ‘of’ should be added between “10%” and “non-alcoholic fatty liver disease”.
  - There needs to be a period at the end of the following sentence “Therefore, reliable noninvasive biomarkers for predicting NASH are required to prevent unnecessary liver biopsies”.
- **Results:**
  - In figures 1a and b, there were no statistics show between the stages. The authors claim that M2BPGi levels and FIB-4 index can estimate the stage of fibrosis. Can every stage be differentiated using these values? To address this, the authors should show if the values are statistically different between stages.
  - In figure 2, the authors should provide a possible explanation to why values were lower for scores 6 and 7 of NAS. Where they statistically lower? Showing statistics or stating the statistics would be helpful.
  - The axes on the figures need to be properly labeled. For example, Figure 1 needs an x-axis label, and the y-axis should be labeled “M2BPGi (COI)”
- **Discussion:**
  - The authors should address their recommendation for if a patient tests positive for one biomarker and not the other. Should the patient get a liver biopsy? Even though the two biomarkers are more accurate together, are they still accurate enough of their own?
